# Repairing sciatic nerve injury with self-assembling peptide nanofiber scaffold-containing chitosan conduit

**DOI:** 10.3389/fneur.2022.867711

**Published:** 2022-10-13

**Authors:** Xuezhen Shen, Feng Qu, Yilun Pei, Simeng Lei, Suhang Xia, Jing Liang, Shan Li, Xun Sun, Liang Liu

**Affiliations:** ^1^Department of Orthopedics, Beijing Luhe Hospital, Affiliated to Capital Medical University, Beijing, China; ^2^Foot and Ankle Surgery Center, Beijing Tongren Hospital, Affiliated to Capital Medical University, Beijing, China; ^3^Department of Pediatrics, Tianjin Hospital, Tianjin University, Tianjin, China; ^4^Department of Orthopedics, Tianjin Hospital, Tianjin University, Tianjin, China

**Keywords:** peripheral nerve defects, nerve regeneration, nerve guidance conduits, combined therapy, self-assembling peptide

## Abstract

**Background:**

An increasing number of nerve guide scaffolds have been used to replace the “gold-standard” autologous nerve graft for repairing peripheral nerve defects, but nerve regeneration is usually far from complete.

**Methods:**

Here, we designed and prepared two functionalized self-assembling peptides (SAP) with the IKVAV and KLT sequences, which were derived from the combination of laminin and VEGF, respectively. Their mixtures were also obtained to combine the effects of neuroprotective and neurotrophic and proangiogenic factors.

**Results:**

The beneficial effect of peptide gels on nerve regeneration was evaluated *in vitro* using Schwann cells (SCs). As a useful intraluminal filling, a three-dimensional (3D) functionalized self-assembling peptide (SAP) nanofiber hydrogel was formed in the hollow lumen of chitosan conduits under physiological conditions. *In vivo*, the combination of the two functionalized SAP gels containing a chitosan nerve conduit significantly accelerated nerve healing and enhanced morphological repair.

**Conclusion:**

Based on the current findings, the combined application of two functionalized SAP gels with chitosan nerve conduit is a promising therapy for the engineering of peripheral nerve regeneration.

## Introduction

Peripheral nerve injury (PNI) leads to substantial motor and sensory deficits (1, 2). The peripheral nervous system (PNS) has the ability to self-repair small injuries. However, nerve grafts must be used for more substantial defects. Autologous nerve grafting is the gold standard method for repairing peripheral nerve defects with a gap, but it also has several disadvantages: these include the limited number of available grafts, donor site morbidity, and risk of neuroma (3). Therefore, artificial nerve guide conduits (NGCSs) have been developed to bridge gaps between severed peripheral nerve stumps and promote nerve regeneration (4–6). Unfortunately, the therapeutic effect of most commercial NGCSs is limited because of their simple constructs (7, 8). The ideal NGCS must have good biocompatibility and biochemical cues sufficiently similar to the native extracellular matrix (ECM) of nerve tissues to promote a favorable microenvironment for nerve regeneration.

In the medical science field, the SAPs have widely been used by forming membranes or hydrogels. Depending on the formation style, peptide hydrogels can also be classified as chemically crosslinked peptide hydrogels and physically crosslinked peptide hydrogels (9). Physical peptide hydrogel is formed by intra- and intermolecular self-assembly, which is generated by non-covalent interactions, such as hydrogen bonding, electrostatic interactions, hydrophobic interactions (10), and Π-stacking (11). According to some researchers, physical peptide hydrogels are self-assembling peptide-based (SAP) hydrogels.

SAP-based biomaterials are effective scaffolds for neural tissue engineering (12–14). RADA16-I, which comprises a regular repeat of positive and negative L-amino acids, is the most extensively studied SAP. Under physiological conditions, RADA16-I forms a highly stable β-sheet structure and self-assembles it into 10-nm-diameter nanofibers. The resulting peptide hydrogel provides a suitable 3D microenvironment for neural cells by mimicking the features of the ECM. Various bioactive peptide motifs can be designed and appended to the C-terminus of RADA16-I by solid-phase synthesis, resulting in SAPs with useful biological activities (15, 16). In a previous study, Zhan et al. first showed that the pure RADA16-I gel filled with an aorta canal segment to bridge a 10-mm sciatic nerve defect (17). Sun et al. demonstrated that a designer SAP hydrogel comprising RADA16-Ile-Lys-Val-Ala-Val (IKVAV) and RADA16-RGD, and having a poly-L-(lactic acid) electrospun conduit, significantly enhanced axonal regeneration and remyelination when used to repair a 5 mm gap in the sciatic nerve defect model (18).

IKVAV (Ile-Lys-Val-Ala-Val) is a laminin sequence that promotes neural adhesion and differentiation and neurite outgrowth (19–21). In this study, we linked the IKVAV sequence to the C-terminus of RADA16-I and assessed the suitability of this functionalized SAP for nerve regeneration. In addition, recent research by Cattin et al. demonstrated that repairing a cut peripheral nerve required blood vessels is used as tracks to guide Schwann cell migration at injury sites, and vascular endothelial growth factor (VEGF) could arouse the polarized vascularization of the bridge region (22). Then, we choose the angiogenic motif KLT (KLTWQELYQLKYKGI), which is a VEGF-mimicking peptide and could bind to and activate the VEGF receptors (23). Previous reports demonstrated that the functionalized SAP RAD/KLT gel's effectivity enhanced endothelial cell survival, migration *in vitro*, and induce new capillary vessel formation *in vivo* (24, 25). In summary, we synthesized two functionalized SAPs RAD/KLT (Ac-(RADA)_4_-GG-KLTWQELYQLKYKGI-NH_2_) and RAD/IKVAV (Ac-(RADA)_4_-GG-IKVAV-NH_2_), which have a promoting effect on nerve tissues or blood vessel, respectively. By mixing these two functionalized SAPs, their mixtures RAD/KLT/IKVAV were obtained to create synergies.

In this study, the designer SAP scaffold hydrogel RAD/KLT, RAD/IKVAV, and their mixtures RAD/KLT/IKVAV were first tested *in vitro* using Schwann cells (SCs). We proposed the use of the natural conduits based on chitosan for repairing sciatic nerve defects because not only does chitosan has appealing properties such as cytocompatibility and biodegradability (26), but its main degradation product chitooligosaccharides (COS) can also promote the proliferation and growth of cells (27, 28). So they were then used as intraluminal fillings within hollow chitosan conducts to bridge 10 mm-long sciatic nerve gaps in rats. We hypothesized that the combined therapy of proangiogenic factors and neuroprotective and neurotrophic roles could further promote peripheral nerve regeneration, compared with using a single strategy.

## Materials and methods

### Synthesis of the designer SAP and scaffold preparation

**Figure 1:**
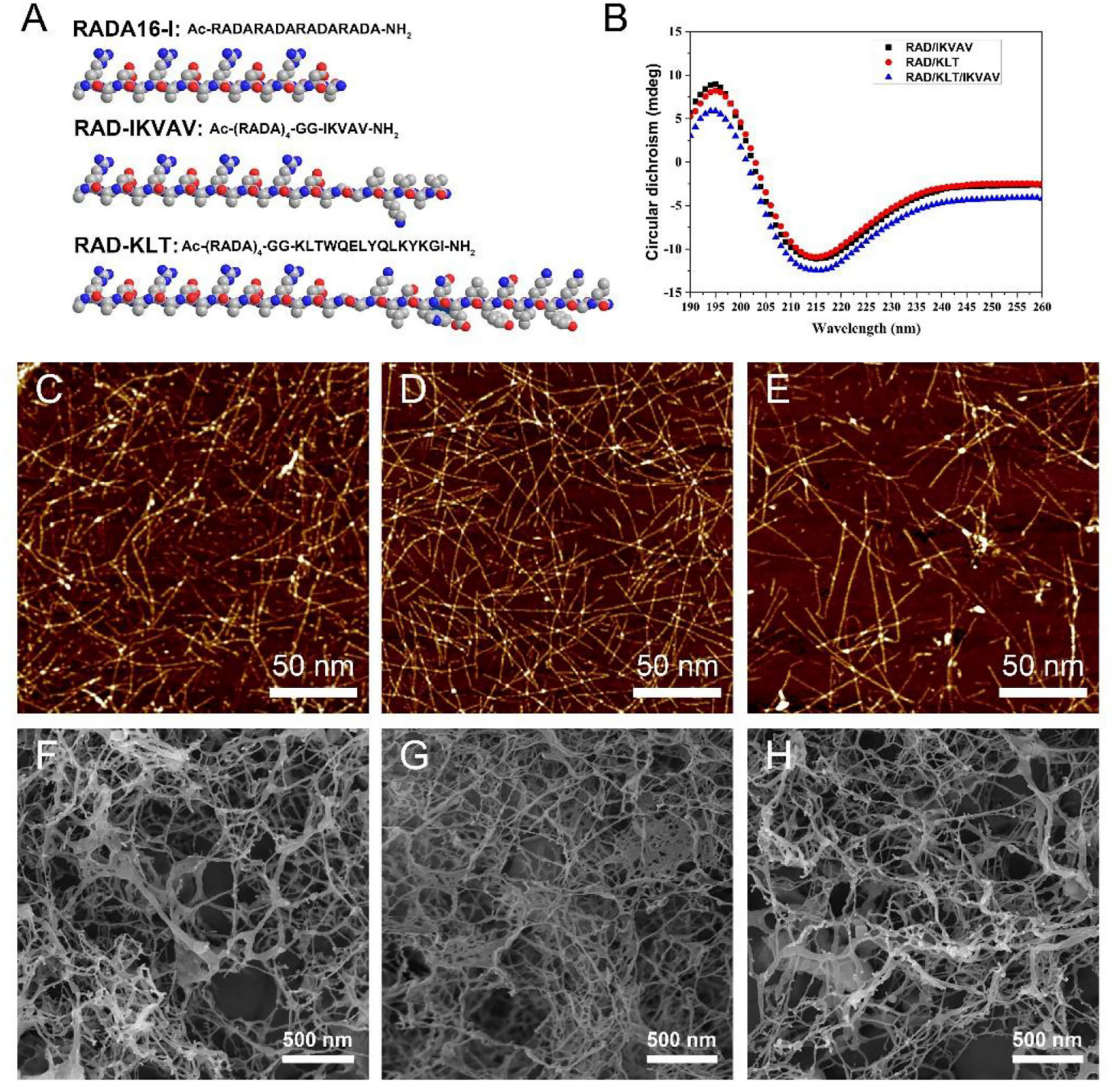
**(A)** The chemical structure and space-filling model of RADA16-I and the functional SAP RAD-IKVAV and RAD-KLT. **(B)** CD spectra of different SAPs mixtures RAD/KLT, RAD/IKVAV and RAD/KLT/IKVAV. The AFM images of RAD/KLT. **(C)**, RAD/IKVAV **(D)**, and RAD/KLT/IKVAV **(E)**. The SEM images of RAD/KLT **(F)**, RAD/IKVAV **(G)**, and RAD/KLT/IKVAV **(H)**.

All the peptides (purity > 90%) used in this work were custom-synthesized and purified by the ChinaPeptides Co., Ltd (Shanghai, China). The peptide sequences are shown in Figure 1A. These peptide powders were dissolved in distilled water at a final concentration of 1% (w/v, 10 mg/mL) using 15 min of sonication (VCX 130PB, Sonics, CT). All of the peptide solutions were filter-sterilized with an Acrodisc Syringe Filter (0.2 μm HT Tuffrun membrane, Pall Crop., Ann Arbor, MI) prior to their use. The functionalized peptide solutions were mixed with 1% pure RADA16-I solution with a volume ratio of 1:1 to obtain functionalized peptide mixtures RAD/KLT and RAD/IKVAV or with a volume ratio of 2:1:1 to obtain functionalized peptide mixtures RAD/KLT/IKVAV.

Cell culture transwell inserts (10 mm diameter, Millipore, MA) were used for the preparation of the SAP hydrogels, as previously described (29). Briefly, the sterilized insets were placed in a 24-well culture plate, and 400 μL of basal culture medium (Dulbecco's modified Eagle's medium and Ham's F12 medium, DMEM/F12, Hyclone) was added to each well. After removing the cell culture medium, 100 μL of peptide solution (RAD/KLT, RAD/IKVAV, or RAD/KLT/IKVAV) was then added, and the plate was incubated at 37°C for 15 min to allow gelation to occur. Subsequently, 400 μL of culture medium was gently loaded onto the gel and the plate was then incubated at 37°C for another 15 min. The whole medium in the well was changed at least three times to equilibrate the hydrogel to physiological pH and then incubated overnight at 37°C for further use. Then, the medium was changed every 2 days.

### Circular dichroism (CD)

The secondary structure of the peptide was measured by the CD spectra. One percent peptide solutions were diluted with water to a working concentration of 0.01%. An Applied Photophysics Chirascan instrument was used with a 1 mm pathlength quartz cuvette to collect scans from 190 to 260 nm of 400 μL of samples. Each spectrum was collected in triplicate.

### Atomic force microscopy (AFM)

The 1% RAD/KLT, RAD/IKVAV, or RAD/KLT/IKVAV solutions were diluted to a concentration of 0.01%. The diluted samples were dropped onto a freshly cleaved mica surface for 30 s and then rinsed with 200 μL of distilled water. After dried in the air, the peptide samples on the mica were immediately observed by AFM (Dimension 3100 SPM, Bruker, Germany), with a scanning area of 2 × 2 μm and scan frequency of 1.00 Hz.

### Scanning electron microscopy (SEM)

After gelation, the SAP nanofiber hydrogel was fixed with 2.5% glutaraldehyde for 2 h. After fixation, samples were washed twice in PBS and subsequently dehydrated in successive ethanol washes. Once dehydrated, samples were dried through CO_2_ critical point drying (Samdri-PVT-3D, Tousimis, USA). The fresh fracture surfaces of the samples were sputter-coated with a layer of Pt for scanning electronic microscope (SEM, ZEISS MERLIN VP compact, Germany) imaging.

### Proliferation and differentiation of schwann cells (SCs)

SCs were harvested from the sciatic nerves of 3-day-old SD rats as described previously (30). The rats were obtained from the Experimental Animal Center of the Chinese PLA general hospital, and the experiments were performed in accordance with the Guides for the Care and Use of Laboratory Animals from the Chinese Ministry of Public Health and the US National Institutes of Health. And the study was reviewed and approved by the Animal Experiments and Experimental Animal Welfare Committee of Capital Medical University. Primary cultures of SCs were maintained in DMEM/F12 medium supplemented with 10% fetal bovine serum at 37°C under humidified 5% CO_2_. After 48 h, the supernatant was removed and SCs were resuspended in 1 mg ml^−1^ collagenase NB4 to purify from fibroblasts. The purified SCs were seeded on the surface of various peptide gels prepared as described above at a density of 1 × 10^6^ cells/mL for 3 days. The gel-free group was used as normal control. Following the cell culture, the cells were fixed with 4% paraformaldehyde for 2 h and permeabilized with 0.1% Triton X-100 for 5 min and 0.1% BSA for 30 min at room temperature. Then, the samples were stained overnight at 4°C with the primary antibody Myelin Protein Zero (P0, 1:250, NB100-1607, NOVUS) and S100 (1:100, ab52642, Abcam), followed by the secondary antibody Alexa 488 (1:200, ab150169, Abcam) and Alexa 594 (1:200, ab150084, Abcam) for 2 h. After washing three times with PBS, the samples were stained with 40, 6-diamidino-2-phenylindole (DAPI) for 10 min and then washed three times with water. All the stained samples were visualized by laser confocal scanning microscopy (Leica, Germany). The rate of P0 and S100 double-positive cells in the various peptide gels groups and gels-free group (Normal group) were calculated according to 10 randomly selected maximum projection fields of each group at 200 × magnification using IPP 6.0.

A total of 50 μL of various peptide solutions were loaded directly into the wells of a 96-well plate and then 150 μL of culture medium was added to the peptide solution for gelation. Once the hydrogel formed, the culture medium was carefully removed and changed twice to equilibrate the gels to physiological pH prior to plating the cells. The purified SCs obtained by the above method were seeded 5,000 cells per well on the surface of various peptide gels and gels-free wells (as normal control). After incubation for 1, 3, and 5 days in an incubator at 37°C under an atmosphere of 5% CO2, the medium in each group was replaced with 100 μL per well fresh medium, and the Cell Counting Kit-8 (CCK-8) solution (CK04, Dojindo) was added to each well (10 μL per well). Thereafter, each group of SCs was incubated for 2 h. The absorbance at 450 nm was measured using a trace orifice spectrophotometer (EPOCH TAKE 3, BioTek). The proliferative ability of each group of SCs was defined as A_experimental_ – A_blank_. All measurements were independently repeated three times.

### Surgical procedure

Ethical considerations in animal studies were identical to the previously described in the part of proliferation and differentiation of SCs. A total number of 60 healthy male 8-week-old Sprague-Dawley rats, weighing 200–220 g were obtained from the Experimental Animal Center of the Chinese PLA General Hospital. Prior to implantation, rats were anesthetized *via* intraperitoneal injection of 3% sodium pentobarbital solution (2.5 mg/100 g body weight). As shown in **Figure 3**, all the rats were randomly divided into five groups with 12 rats in each group, including hollow chitosan nerve conduit group (Hollow), chitosan nerve conduit filled with RAD/KLT nanofiber hydrogel (RAD/KLT), RAD/IKVAV nanofiber hydrogel (RAD/IKVAV) or RAD/KLT/IKVAV nanofiber hydrogel (RAD/KLT/IKVAV) group, and autologous nerve graft group (Autograft). In the Hollow group, an empty chitosan tube (CST) was placed between the two ends of the transected nerve. In the RAD/KLT, RAD/IKVAV, and RAD/KLT/IKVAV groups, the grafts were fabricated by filling the RAD/KLT, RAD/RGI, and RAD/KLT/RGI solution into the lumen of a CST. The peptide solutions were first injected into the hollow empty chitosan tubes (CSTs). Gelation of the solutions in the tubes was then achieved by soaking in Dulbecco's modified Eagle's medium (DMEM; Invitrogen, Carlsbad, CA, USA) for 2–3 min followed by PBS for another 5 min.

The sciatic nerve of the right hind was exposed after making a skin incision and splitting the muscles, and a 10-mm transected segment was cut off. Then the gaps were bridged by different chitosan nerve conduits or autologous nerves in different groups. After implantation, the muscle and skin were sutured. After surgery, all rats were housed and fed routinely and monitored for any changes.

### Histology and morphometric evaluation of nerves

Five rats in each group were sacrificed by intraperitoneal injection of a sodium pentobarbital overdose in week 6 after surgery. The implanted grafts were clearly exposed and integrally excised. The transversal surface in the middle of the implanted grafts in each group was rapidly fixed in 4% formaldehyde for 2 h. The grafts were embedded in paraffin and cut into 5 μm thick transversal sections for Masson's trichrome staining using a commercial staining kit (G1345, Solarbio). Ten randomly selected fields of each group at 400 × magnification were captured to count the neovascular count and neovascular area ratio per view field by IPP 6.0.

At week 12 after surgery, the rest of the rats in each group were sacrificed by intraperitoneal injection of an overdose of sodium pentobarbital following electrophysiological evaluation. The regenerative nerves located at the distal ends of the implanted grafts were fixed to cut transverse semi-thin sections at a thickness of 1 μm using a glass knife in an ultramicrotome (EM UC7; Leica) and stained with Toluidine Blue O solution (1% in sodium borate; G3663; Solarbio). The stained sections were observed under 400 × magnification, and 10 randomly selected fields were captured for each group to count the density of myelinated nerve fibers by IPP 6.0.

Subsequently, according to the previous research, the light microscopic estimation of myelinated fibers significantly underestimated the total number compared to the electron microscope measurement, and to reduce the error and analyze the results more accurately, we observe the diameter of myelinated nerve fibers and the thickness of the myelin sheath by transmission electron microscopy (31). The regenerative nerves were cut into transverse ultrathin sections 70 nm thick using a diamond knife in an ultramicrotome (EM UC7; Leica) and collected on copper slot grids with pioloform/carbon support films. The ultrathin sections were counterstained with 3% lead citrate and uranyl acetate, and were observed by transmission electron microscopy (TEM) (CM-120; Philips). Ten randomly selected fields at 5,000 × magnification were captured for each group to measure by IPP 6.0. The method referred to the previously published methods (30, 32).

### Ultrasonography of gastrocnemius

At 6 weeks after surgery, an ultrasonography examination was performed to assess the morphology and elasticity of the gastrocnemius at the injured and contralateral sites (*n* = 5). A scanner of Apolio-80 color ultrasonic diagnostic instrument (Toshiba, Tokyo, Japan) was used, with a linear array probe, a frequency of 5–14 MHz, and a real-time shear wave elastography (SWE) function. Briefly, the surface projection area of the gastrocnemius muscle was fully 2-D ultrasound, and a color Doppler flow imaging was carried out to observe the size, shape, echo of the gastrocnemius, and blood supply conditions. Then the system was switched to SWE mode on continuous excitation mode without pressure from the probe. After images became stable *via* adjusting the sampling frame size, the images were frozen and the mode was changed to the speed propagation graph model. For images with a good timeline, ROI was selected by using the default circle of the instrument and the young modulus value (EI) and standard deviation were recorded automatically by the machine.

### Wet weight analysis and Masson's trichrome staining of gastrocnemius muscle

At 12 weeks after surgery, the rest of the rats in each group were euthanatized and gastrocnemius muscles in the left and right hind limbs were dissected, detached from the bone, and weighed immediately. Following measurement, the muscle belly of the central segment of the gastrocnemius muscle was fixed in 4% paraformaldehyde at 4°C overnight. They were transversely cut into 7-μm-thick paraffin sections and subjected to Masson's trichrome staining. For each sample, images were taken for ten randomly chosen fields with a light microscope and quantitatively analyzed with the Image Pro Plus 6.0 software.

### Electrophysiological evaluation

At 12 weeks post-surgery, rats were anesthetized with sodium phenobarbital and the sciatic nerves were carefully exposed. The electrical stimulation (3 mA) was applied between the proximal and distal nerve stumps. The compound muscle action potentials (CMAPs) were recorded at the target gastrocnemius muscle with a recording electrode (Keypoint 3.02; Denmark). The peak amplitude and proximal latency of CMAPs were determined from the electromyogram.

### Functional analysis of regenerated sciatic nerve

A CatWalk XT 9.0 gait analysis system (Noldus, Wageningen, The Netherlands) was carried out to evaluate the motor functional recovery following sciatic nerve injury after 2, 4, 6, 8, 10, and 12 weeks. The CatWalk analysis and the required training protocol have been described elsewhere (33). Briefly, the rats (*n* = 3/group/time point) were placed on the right side of the runway with a glass surface and black plastic wall, and each run of the rat was captured by a high-speed camera located under the runway. The standing time, contact area, and intensities of the right injured hind paw (RH) and the normal left hind paw (LH) were recorded. Subsequently, the Sciatic Function Index (SFI) and stand/swing time ratio of each rat were calculated by the CatWalk XT 10.5 system according to the measurement results. SFI was calculated using the following formula (34):


$$\begin{array}{l} \text{SFI }=\;\frac{109.5\left(\text{ETS}-\text{NTS}\right)}{\text{NTS}}-\frac{38.3\left(\text{EPL}-\text{NPL}\right)}{\text{NPL}}\\ +\;\frac{13.3\left(\text{EIT}-\text{NIT}\right)}{\text{NIT}}\;-\;8.8 \end{array}$$

where ETS refers to the experimental toe spread; NTS refers to the normal toe spread; EPL refers to the experimental print length; NPL refers to normal print length; EIT refers to experimental inter toe spread and NIT refers to normal inter toe spread.

### Statistical analysis

All the numerical data are presented as means ± standard deviation. The statistical analysis was carried out using the SPSS software version 21.0. Statistical differences between multiple groups were determined using a one-way analysis of variance (ANOVA). Tukey's *post-hoc* test was applied when *p* > 0.05 in the test of homogeneity of variances, otherwise Dunnett's T3 *post-hoc* test was applied. Rank-sum test was applied to analyze ranked data. Differences were deemed to be statistically significant at ^**^ *p* < 0.01 and ^*^ *p* < 0.05.

## Results and discussion

### Characteristics of the designer functionalized SAP

In this study, the designer functionalized peptides RAD-KLT and RAD-IKVAV were synthesized by adding the C-terminus of the self-assembling peptide RADA16-I (Ac-RADARADARADARADA-NH2) to each of the functional motifs, including IKVAV and KLT sequence. The IKVAV sequence was derived from laminin, and the peptide motif KLT was derived from VEGF. Two glycine residues were used as a spacer linker to maintain the flexibility of the functionalized SAP. The chemical structure and space-filling model of RADA16-I and the functional SAP are shown in Figure 1A. To enhance the ability of self-assembly for functionalized peptides, the mixed peptides RAD/KLT and RAD/IKVAV were obtained by mixing the pure RADA16-I solution with RAD-KLT or RAD-IKVAV at a 1:1 volume ratio, respectively. In addition, we also mixed these two functionalized peptides to exert their combined effect on nerve regeneration and named it RAD/KLT/IKVAV.

To investigate their second structures, the CD measurements in pure water (0.01 mg/mL) were performed (Figure 1B). The results showed that all three peptide mixtures indicated a typical β-sheet structure with a minimum ellipticity at ~216 nm and a maximum ellipticity at ~196 nm. Furthermore, the AFM analysis showed that these three functionalized peptides mixtures could form a long uniform self-assembled nanofiber fragment (Figures 1C–E). By exchanging the cell culture medium, they could form hydrogel scaffolds under physiological condition. The SEM images of peptide hydrogels also confirm the self-assembling nanofiber formations. As shown in Figures 1F–H, the interweaved long nanofibers could be observed in all three functionalized peptide mixtures, which suggested that the functionalized peptides interact with the unmodified RADA16-I and incorporate into the nanofibers quite well. And the SEM images of these peptide hydrogels were consistent with the results from the AFM analysis.

### *In vitro* study

### Proliferation and differentiation of schwann cells (SCs)

**Figure 2:**
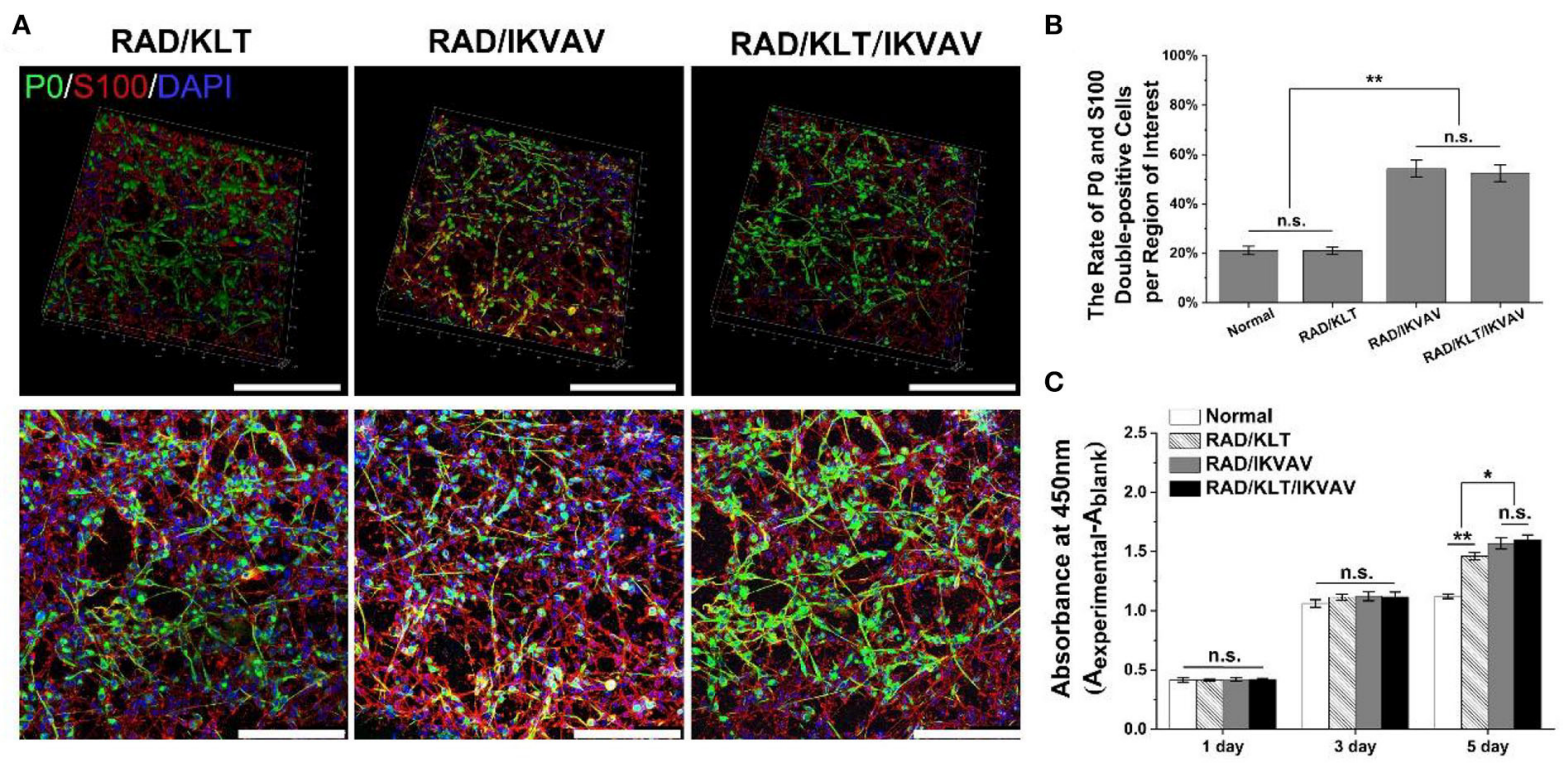
Effect of peptide gels on proliferation and differentiation of Schwann cells. **(A)** Representative immunofluorescence staining images of SCs from each peptide gels. The top row is the 3D views and the bottom row are the maximum projectional views. **(B)** The rate of P0 and S100 double-positive cells per region of interest in each group. **(C)** The CCK-8 assay results of SCs in each groups. Data are expressed as means ± SEM. ^*^p < 0.05, ^**^p < 0.01, n.s. represents no significant difference, one-way ANOVA with Tukey's post-test or Dunnett T3's post-test. Scale bar = 200 μm.

After 3 days of incubation, SCs from each peptide gel showed a narrow fusiform-like shape with a bipolar or tripolar structure, and the SCs in the RAD/IKVAV and RAD/KLT/IKVAV groups seemed to stretch better (Figure 2A). The S100 protein, known as an SCs marker, was labeled with red fluorescence, and the SCs differentiated into myelinated SCs were labeled with green fluorescence by Myelin Protein Zero (P0) staining. The nuclei were shown in blue fluorescence by DAPI staining (Figure 2A). Statistical results showed that a significantly higher percentage of P0 and S100 double-positive cells (*p* < 0.01) were observed in the RAD/IKVAV (54.25 ± 3.45 % per region of interest) and RAD/KLT/IKVAV groups (52.48 ± 3.38 % per region of interest) than that in the RAD/KLT (21.10 ± 1.45 % per region of interest) and Normal groups (21.14 ± 1.73 % per region of interest). However, there were no significant differences between the RAD/IKVAV and the RAD/KLT/IKVAV groups or between the RAD/KLT and the Normal groups (Figure 2B).

The optical density (OD) indirectly represents the number of cells in the CCK-8 assay. SCs from each peptide gel gradually increased in number over time in culture. After 1 day and 3 days of incubation, there were no significant differences among the various peptide gels groups and gels-free group (Normal group). When SCs of each group were incubated for 5 days, the ODs of the RAD/IKVAV group (1.57 ± 0.05) and RAD/KLT/IKVAV group (1.60 ± 0.04) were significantly higher than those of the RAD/KLT group (1.46 ± 0.03, *p* < 0.05) and Normal group (1.12 ± 0.02, *p* < 0.01). In the meantime, the values were significantly different between the RAD/KLT and the Normal groups (*p* < 0.01), and there were no significant differences between the RAD/IKVAV and RAD/KLT/IKVAV groups (Figure 2C).

### Histological analysis of regenerated sciatic nerve

**Figure 3:**
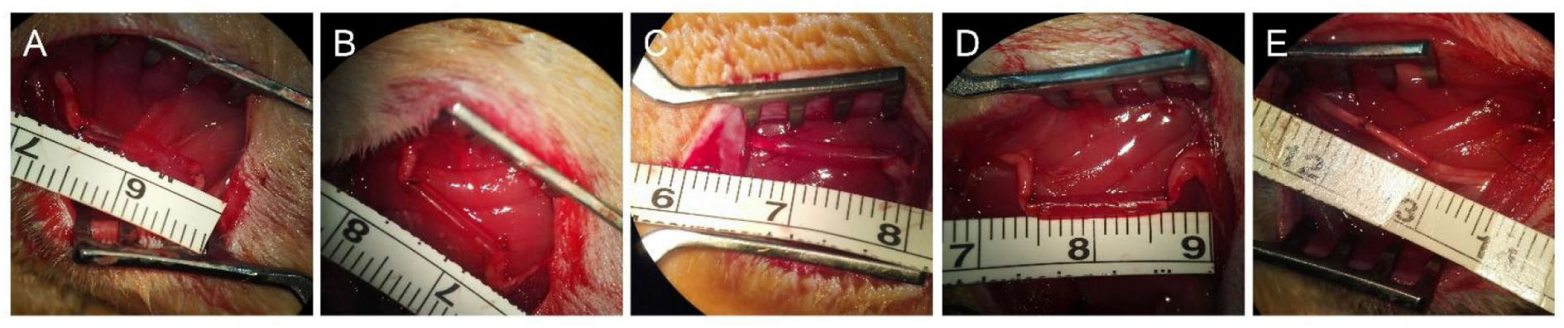
Preparation and implantation of the nerve grafts of different groups. Gross morphologies of hollow chitosan nerve conduit group (Hollow, **A**), chitosan nerve conduit filled with RAD/KLT nanofiber hydrogel (RAD/KLT, **B**), RAD/IKVAV nanofiber hydrogel (RAD/IKVAV, **C**) or RAD/KLT/IKVAV nanofiber hydrogel (RAD/KLT/IKVAV, **D**) group, and autologous nerve graft group (Autograft, **E**).

**Figure 4:**
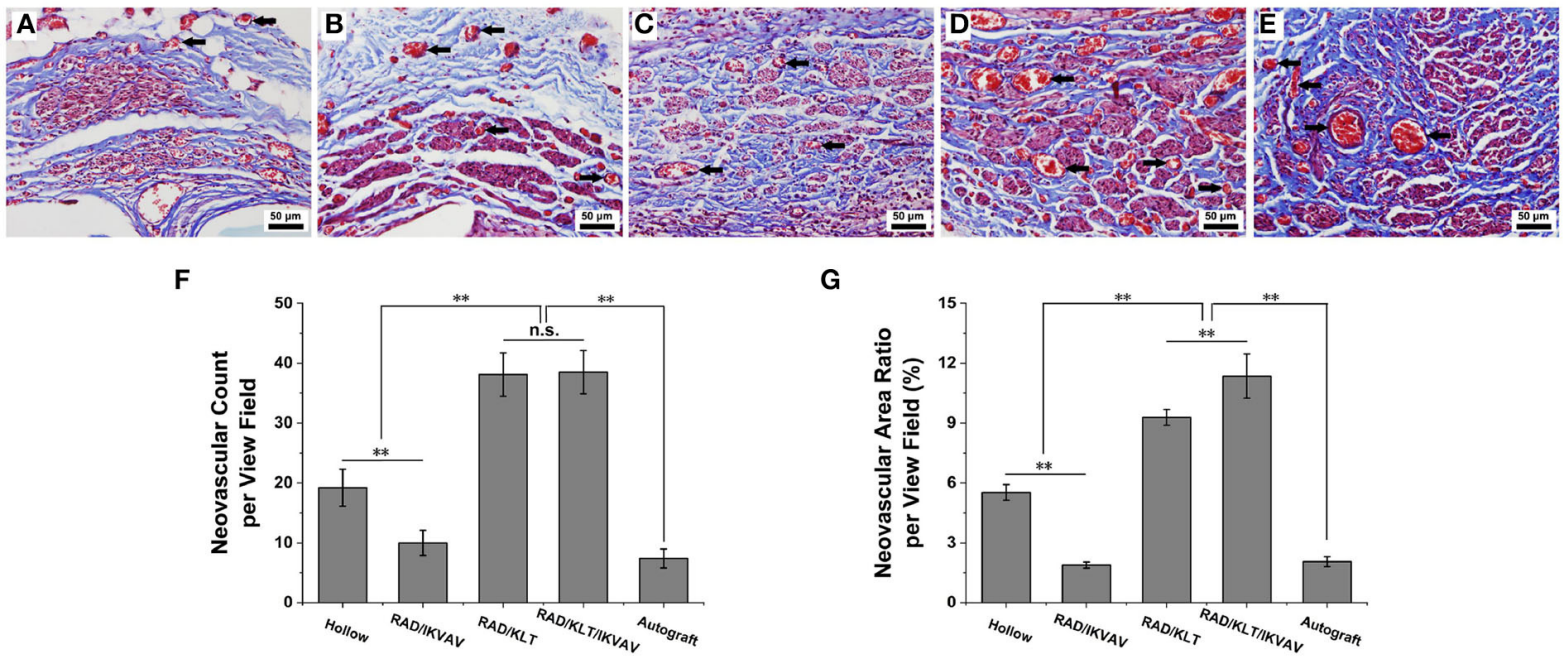
Histological analysis of angiogenesis in the middle segments of the implanted grafts at 6 weeks after surgery and the neovascular of the implanted grafts are indicated by black arrows. **(A–E)** Representative transverse images of Masson's trichrome staining from **(A)** Hollow group **(B)** RAD/IKVAV group **(C)** RAD/KLT group **(D)** RAD/KLT/IKVAV group, and **(E)** Autograft group. **(F)** The neovascular count per view field in each group. **(G)** The neovascular area ratio per view field in each group. Data are expressed as means ± SEM. *p < 0.05, **p < 0.01, n.s. represents no significant difference, one-way ANOVA with Tukey's post-test or Dunnett T3's post-test.

Representative dissection photographs of the grafting segment in each group are shown in Figure 3. To investigate the effect of self-assembling peptide nanofiber peptide scaffolds on nerve regeneration and angiogenesis *in vivo*, the regenerated nerves of all groups were harvested at predetermined time points. Masson's trichrome staining revealed the transverse section of the implanted grafts in the middle segments 6 weeks post-implantation. As shown in Figures 4A–E, the RAD/KLT and RAD/KLT/IKVAV groups showed the highest level of neovascular, followed by the Hollow group, and the lowest neovascular density in the RAD/IKVAV and Autograft groups. The quantitative analysis of neovascular count and area ratio was further determined (Figures 4F–G). The neovascular count and neovascular area ratio per view field showed similar trends, i.e., those in the RAD/KLT (38.1 ± 3.6 in count and 9.3 ± 0.4% in area ratio) and the RAD/KLT/IKVAV groups (38.5 ± 3.6 in count and 11.4 ± 1.1% in area ratio) were significantly higher (*p* < 0.01) than those in the Hollow (19.2 ± 3.1 in count and 5.5 ± 0.4% in area ratio), RAD/IKVAV (10.0 ± 2.1 in count and 1.9 ± 0.2% in area ratio), and Autograft groups (7.4 ± 1.6 in count and 2.1 ± 0.3% in area ratio). Interestingly, there were no significant differences in neovascular count per view field between the RAD/KLT and RAD/KLT/IKVAV groups, but the neovascular area ratio per view field in the RAD/KLT/IKVAV group was significantly higher (*p* < 0.01) than that in the RAD/KLT group.

**Figure 5:**
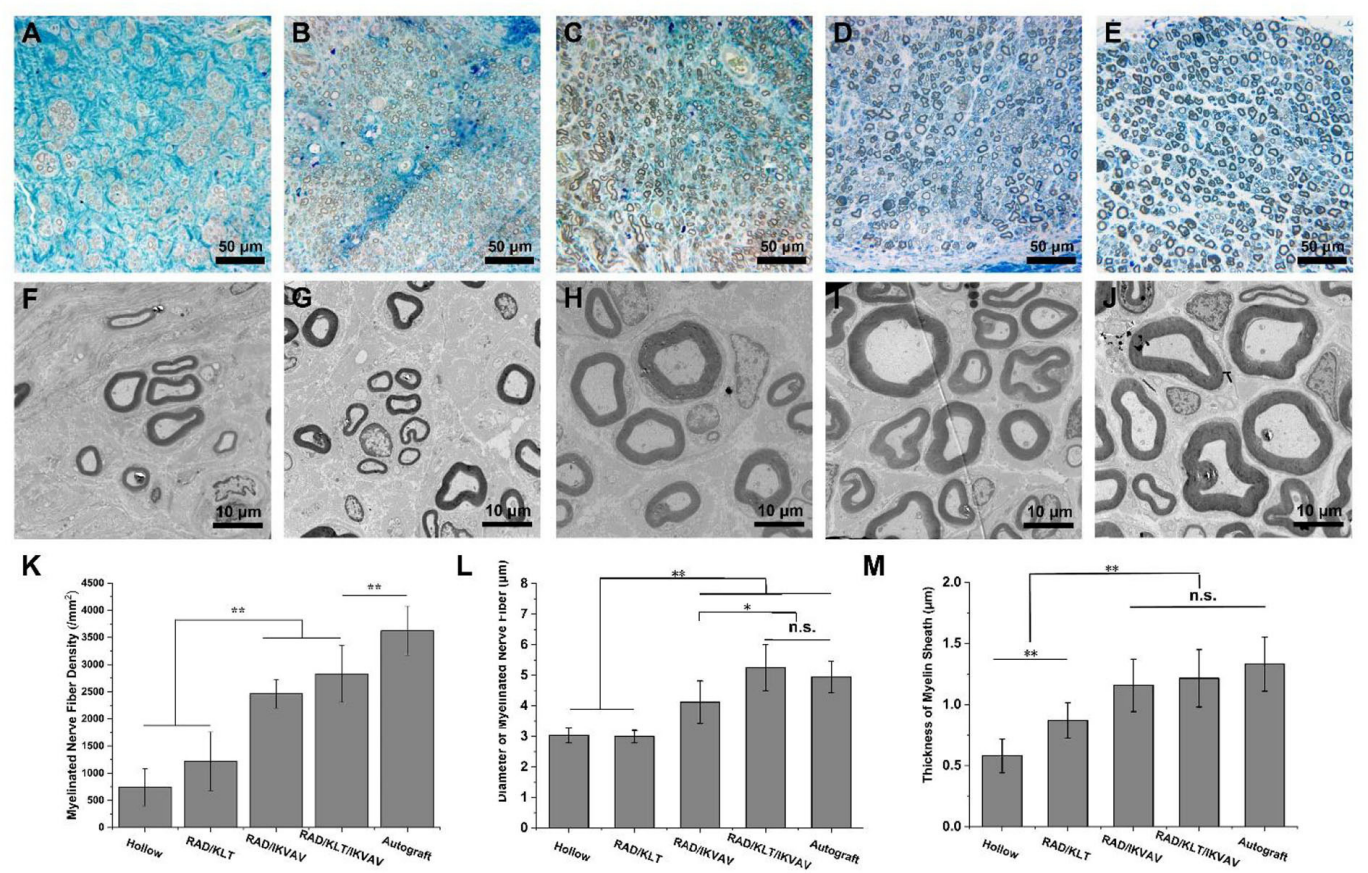
Morphometric analysis of middle portions of sciatic nerves at 12 weeks after the operation. **(A–E)** Representative images of semi-thin transverse sciatic nerve sections from **(A)** Hollow group **(B)** RAD/KLT group **(C)** RAD/IKVAV group **(D)** RAD/KLT/IKVAV group, and **(E)** Autograft group **(F–J)** Representative images of ultra-thin transverse sciatic nerve sections from **(F)** Hollow group **(G)** RAD/KLT group **(H)** RAD/IKVAV group, **(I)** RAD/KLT/IKVAV group, and **(J)** Autograft group. **(K)** The density of myelinated axons is expressed as the number of myelinated axons per square millimeter (n/mm^2^). **(L)** The diameters of myelinated axons. **(M)** The thickness of myelin sheath. Values represent mean ± SD; n.s. no significant difference, **p < 0.01, *p < 0.05 in multiple comparisons between treatment groups.

To assess the re-myelination extent after injury, transverse semi-thin sections of the regenerated nerve fibers were analyzed by toluidine blue staining (Figures 5A–E). The results showed that uniform myelinated nerve fibers were formed in all of the sample groups. However, the hollow group exhibited thinner myelin sheaths and less myelinated axons than other groups (Figure 5A). Large numbers of myelinated axons and thick myelin sheaths were observed in the RAD/KLT/IKVAV and Autograft groups (Figures 5D–E).

In addition, the density of myelinated axons in RAD/IKVAV (2461.0 ± 262.8 /mm^2^) and RAD/KLT/IKVAV (2829.9 ± 525.9 /mm^2^) groups were significantly higher than the Hollow (739.0 ± 347.9 /mm^2^) and RAD/KLT (1218.0 ± 539.5 /mm^2^) group, although they could not catch the Autograft (3623.4 ± 452.6 /mm^2^) group (Figure 5K). We also evaluated the extent of the regeneration of myelinated axons *via* TEM (Figures 5F–J). The TEM morphologies displayed thicker packed nerve fibers in the RAD/IKVAV, RAD/KLT/IKVAV, and Autograft groups than in the Hollow and RAD/KLT groups. The statistical analysis confirmed that the diameter of the myelinated nerve fiber in the RAD/KLT/IKVAV (5.2 ± 0.8 μm) group was larger than the other three groups, which was slightly higher than the Autograft (4.9 ± 0.5 μm) group (Figure 5L). In addition, there was no significant difference between the RAD/IKVAV, RAD/KLT/IKVAV, and Autograft groups in terms of the thickness of the myelin sheath. They showed better re-myelination of the regenerated nerves than the Hollow and RAD/KLT groups (Figure 5M).

### Electrophysiological analysis

**Figure 6:**
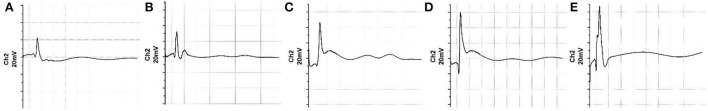
Electromyograph of the nerve regeneration at 12 weeks post-implantation. **(A)** Hollow group. **(B)** RAD/KLT group. **(C)** RAD/IKVAV group. **(D)** RAD/KLT/IKVAV group, and **(E)** Autograft group.

**Figure 7:**
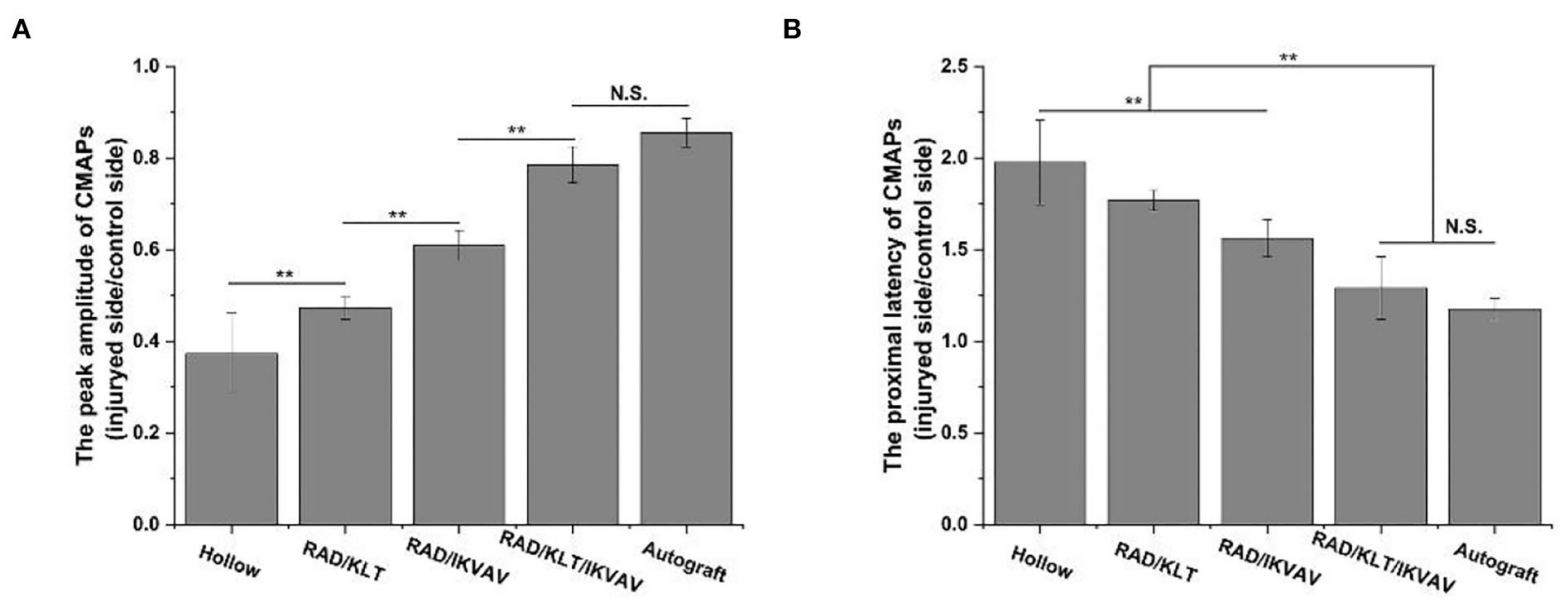
**(A)** The peak amplitude of CMAPs of the regenerated nerves. **(B)** The proximal latency of CMAPs. Values represent mean ± SD; N.S. no significant difference, **p < 0.01, *p < 0.05 in multiple comparisons between treatment groups.

The electromyographic examination results of gastrocnemius muscle at 12 weeks after surgery are shown in Figure 6. The peak amplitude of CMAPs in the RAD/KLT/IKVAV group (78.53 ± 3.94%) was significantly higher than that in the Hollow (37.34 ± 8.81%), RAD/KLT (47.33 ± 2.49%), and RAD/IKVAV groups (60.90 ± 3.29%), which was similar to the Autograft group (85.59 ± 3.15%). And the peak amplitude of CMAPs in the RAD/IKVAV group was significantly higher than that in the RAD/KLT group (*p* < 0.01; Figure 7A). In addition, the proximal latency of CMAPs in the RAD/KLT/IKVAV(1.29 ± 0.17) and Autograft group (1.17 ± 0.06) were shorter than that in the other three groups. There was no significant difference among the Hollow (1.98 ± 0.23), RAD/KLT (1.77 ± 0.56), and RAD/IKVAV (1.56 ± 0.10) groups in terms of the proximal latency of CMAPs. The latency ratio of CMAPs was not significantly different among the Autograft group and RAD/KLT/IKVAV group (*p* ≥ 0.05), and the ratios of the other three groups were significantly higher than that of Autograft and RAD/KLT/IKVAV group (*p* < 0.01; Figure 7B).

### Reinnervation of target gastrocnemius muscle

**Figure 8:**
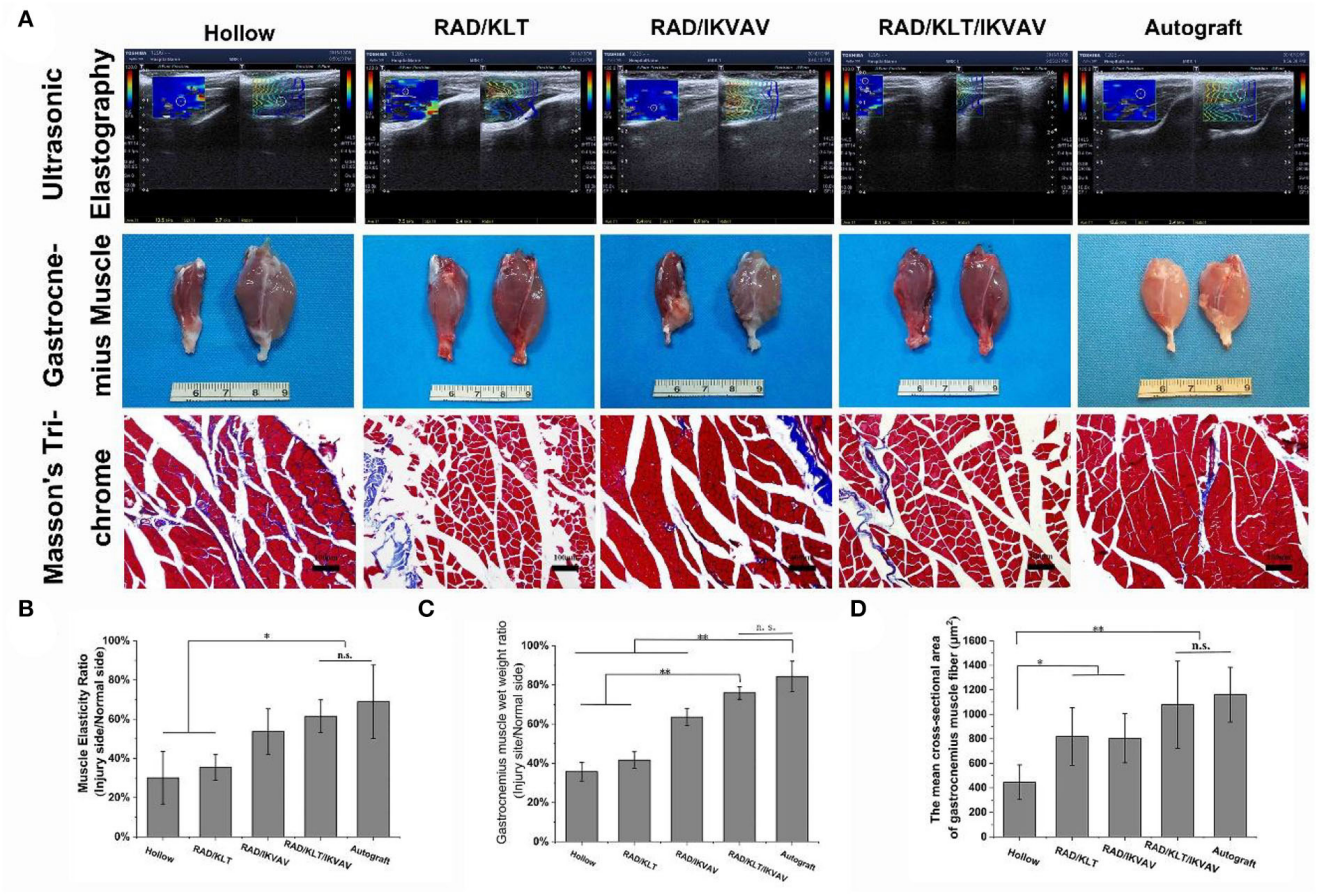
**(A)** Ultrasonography of gastrocnemius at 6 weeks after surgery. Gross images of the isolated gastrocnemius muscles and the Masson's trichrome staining images of the transverse sections of muscles from the injured limbs at 12 weeks after implantation. Statistical analysis of the muscle elasticity ratio **(B)**, the gastrocnemius muscle wet weight ratio **(C)**, and the percentage of muscle fiber area **(D)**. Values represent mean ± SD; n.s. no significant difference, **p < 0.01, *p < 0.05 in multiple comparisons between treatment groups.

At 6 weeks after surgery, we first examined the gastrocnemius recovery using ultrasound imaging (Figure 8A). The muscle elasticity ratio was calculated according to the ultrasound imaging. As shown in Figure 8B, the muscle elasticity ratio in the RAD/KLT/IKVAV (61.53 ± 83.13) and Autograft groups (68.86 ± 18.74) were significantly higher than that in the Hollow (30.1 ± 13.55), RAD/KLT(35.41 ± 6.58), and RAD/IKVAV (53.71 ± 11.55) groups. And there was no significant difference between the RAD/KLT/IKVAV and Autograft group. The macroscopic views of gastrocnemius muscles from the injury site and normal site in all groups at 12 weeks after surgery are shown in Figure 8A. The atrophy of gastrocnemius muscle was obviously seen in the Hollow, RAD/KLT, and RAD/IKVAV groups, which was not found in the RAD/KLT/IKVAV and Autograft groups. Like the Autograft group (84.30 ± 7.81%), the wet weight ratio of the gastrocnemius muscle in the RAD/KLT/IKVAV group (75.87 ± 3.26%) was significantly higher than that in the other three groups: the Hollow (33.7 ± 4.78%), RAD/KLT (41.67 ± 4.22%), and RAD/IKVAV (63.55 ± 4.09%) (Figure 8C). We also examined the morphological changes in the gastrocnemius muscle using Masson's trichrome staining. And the percentage of muscle fiber area was calculated. As shown in Figure 8D, the percentage of muscle fiber area in the RAD/KLT/IKVAV group (1078.70 ± 355.07 μm^2^) was larger than the Hollow (445.71 ± 141.55 μm^2^), RAD/KLT (819.34 ± 235.97 μm^2^), and RAD/IKVAV groups (804.37 ± 200.64 μm^2^), which was similar to the Autograft group (1159.86 ± 223.50 μm^2^, *p* > 0.05; Figure 8D).

### Cat Walk gait analysis

**Figure 9:**
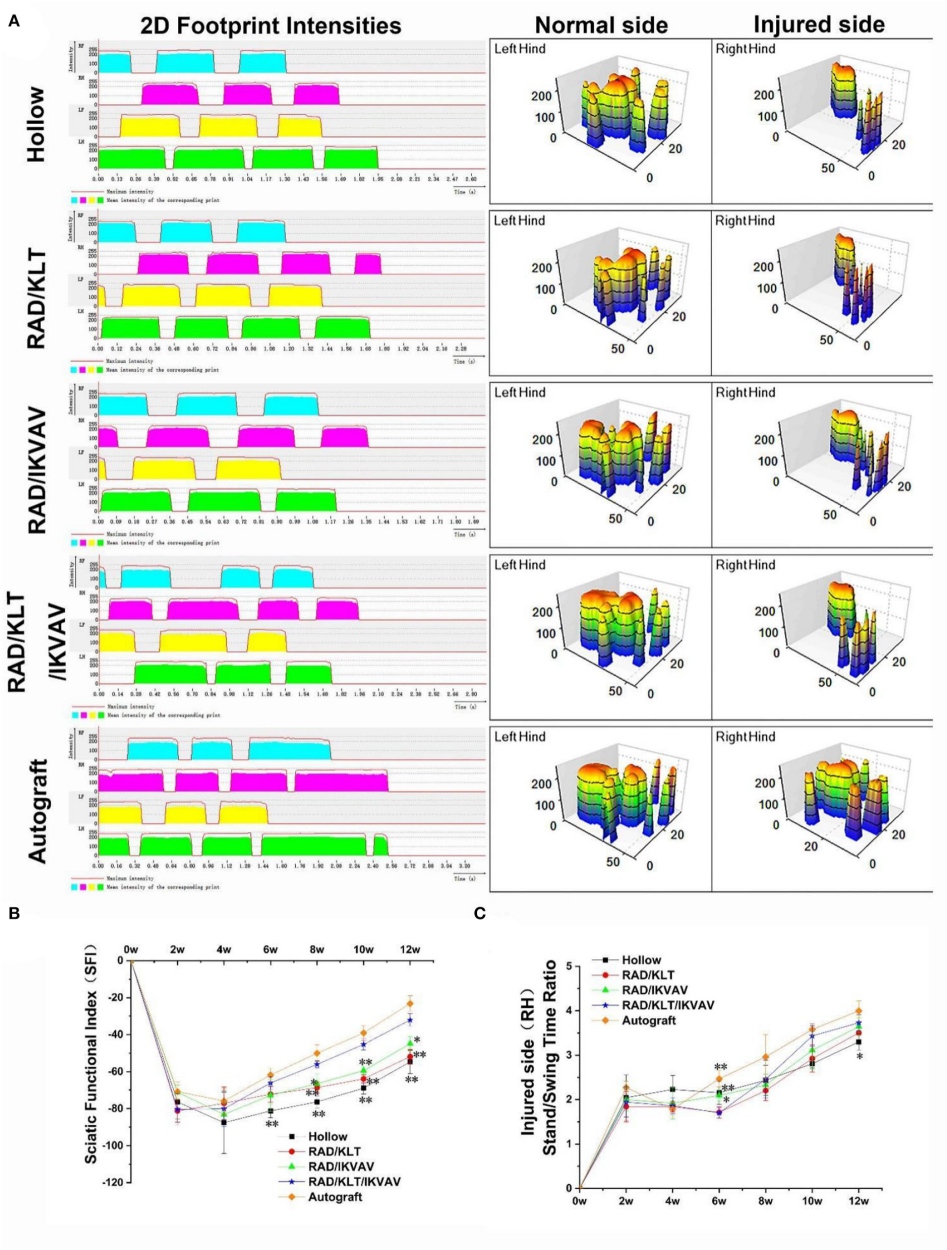
Motor functional recovery at 12 weeks after implantation examined by CatWalk gait analysis. **(A)** Representative CatWalk analysis trace and 3D plantar pressure distribution of LH (contralateral) and RH (injured) from 5 different groups. LF, left front limb; LH, left hind limb; RF, right front limb; RH, right hind limb. Statistical analysis of the SFI values **(B)** and the stand/swing time ratio **(C)**. (*n* = 5 approved runs for each group per time point). Data are expressed as means ± SEM. *p < 0.05, **p < 0.01, one-way ANOVA with Tukey's post-test or Dunnett T3's post-test.

The recovery of motor function was evaluated by the CatWalk gait analysis system (Figure 9). The results of the walking record including the 2D footprint intensities and 3D plantar pressure distribution are shown in Figure 9A. The RAD/KLT/IKVAV group was similar to those of the RAD/IKVAV group. And the Autograft group showed the best rehabilitation in all groups. The sciatic function index (SFI) was then calculated to evaluate the functional recovery of the regenerated nerves at 2, 4, 6, 8, 10, and 12 weeks after surgery. As shown in Figure 9B, the value of SFI in all groups increased with increasing implantation time. For the Autograft group, the SFI was higher than the other four groups at each time point during the whole period. Notably, there was no significant difference between RAD/IKVAV (−72.20 ± 4.21)and RAD/KLT/IKVAV (−66.11 ± 3.38)groups in the initial 6 weeks. However, the SFI of the RAD/KLT/IKVAV group (−56.06 ± 1.94)was higher than the Hollow_(−76.30 ± 3.23), RAD/KLT (−68.65 ± 2.90), and RAD/IKVAV (−66.50 ± 5.25) groups from the 8 weeks after surgery. We also calculated the injured side stand/swing time ratio of all groups as another indicator of motor function recovery (Figure 9C). The stand/swing time ratio of the injury site in the RAD/KLT/IKVAV group (2.46 ± 0.43) was higher than that in the Hollow (2.44 ± 0.34), RAD/KLT (2.20 ± 0.23), and RAD/IKVAV (2.33 ± 0.24) groups from the 8 weeks after surgery, although it was less than that in the Autograft group (2.96 ± 0.50).

## Discussion

Nerve defects and injuries are serious problems of clinical disease. It is reported that approximately 13–23 per 100,000 persons annually suffer from intensive peripheral nerve injuries, and up to one-third of the patients may have little or no recovery whatsoever despite appropriate surgical intervention (35, 36). It is followed by a huge economic burden on patients and society (37). Fortunately, there are multiple studies currently pursuing improved repair of peripheral nerve injury. It has recently been suggested that promoting angiogenesis at the injury site and the IKVAV could promote nerve regeneration, which may be useful in the treatment of damaged peripheral nerves.

Compared with KLT alone, IKVAV peptide gels can significantly promote myelination and proliferation of SCs *in vitro* (Figure 2), which is consistent with the function of the macromolecular protein derived into IKVAV. IKVAV, a functional peptide derived from Laminin, can promote the adhesion, proliferation, and myelination of glial cells (38). On day 5 of incubation in each peptide gel, the number of SCs was significantly higher than that in the gels-free group (Normal group), while the number of SCs in the Normal group was almost unchanged on days 3 and 5 of incubation. It was related to the saturation of cell density in the Normal group and the thickness of the peptide gels. The SCs on the peptide gels were equivalent to a 2.5 D culture.

We previously developed VEGF mimicking peptide-functionalized SAP nanofiber hydrogel RAD/KLT that produces a neurotrophic microenvironment for improving axon regeneration and motor functional recovery, but its regenerative effect did not catch up with the Autograft group (39). In this study, we expanded our previous efforts to demonstrate the combination therapy of functionalized SAP hydrogel RAD/KLT/IKVAV dual-presenting VEGF- and IKVKA-mimetic peptide epitopes.

In addition, the RAD/KLT and RAD/IKVAV hydrogel improved axon regeneration, innervation of muscles, and recovery of motor function compared with the Hollow group, but all these regenerative effects were significantly less than that of the RAD/KLT/ IKVAV and Autograft groups. The RAD/KLT/IKVAV peptide hydrogel led to parallel angiogenesis and nerve regeneration, which exerted a synergistic effect on peripheral nerve regeneration. Moreover, the injured nerves of the RAD/ KLT/ IKVAV group were recovered more close to the Autograft group, according to morphometric parameters (Figure 5), electrophysiological measurement, and motor functional assessment (Figures 6, 7), innervated muscle weight and remodeling of muscle fibers (Figure 8), motor function (Figure 9).

While neural guidance conduits are promising candidates for bridging small nerve gaps, more aptly designed biomaterials that reduce fibrosis are needed to address larger peripheral nerve injuries. Extensive fibrotic scar tissue can form at an injury site before neurites and glial cells can rebuild neural tissue and this fibrosis can prevent full-functional recovery for larger injuries and may result in debilitating neuromas (40–42).

One important requirement for viable neural guidance conduits is their ability to impede fibroblast infiltration. In a previous study, a high quantity of nanoscale IKVAV-capped dendrimers incorporated onto pre-crosslinked collagen films promoted rat Schwann cell attachment and proliferation and inhibited human dermal fibroblast proliferation. (43) Angiogenesis at the site of nerve injury is equally important to the repair of nerve injury. Inadequate blood supply is a key issue in tissue-engineered nerve grafts when bridging critical long nerve defects. Insufficient blood supply can lead to more severe inflammation and oxidative stress, making axon regeneration more difficult (44, 45).

In summary, our results suggest that encouraging and imitating the neurovascular microenvironment in the nerve graft and inhibiting the fibrosis in the process of nerve repair may contribute to the process of nerve regeneration. Biochemical signals such as angiogenesis and inhibition of scar formation produced by RAD/KLT/IKVAV polypeptide stents from the microenvironment could regulate and guide the behavior of nerves and improve the recovery of peripheral nerves.

## Conclusion

In this study, two functionalized SAPs were designed and fabricated with the IKVAV and KLT sequence, which were derived from the combination of laminin and VEGF, respectively. To combine the effects of neuroprotective and neurotrophic and proangiogenic factors, their mixtures were also obtained. The functionalized SAP solutions could be injected into the hollow lumen of chitosan conduit and 3D nanofiber hydrogel was formed under the physiological conditions. Our results demonstrate that Schwann cells could adhere to and proliferate on the surface of peptide gels, with good cell compatibility. *In vivo* testing revealed the functional recovery in the RAD/KLT/IKVAV group and autologous graft group were significantly faster than in the hollow conduct group and the other two SAP gels group alone. Histological examinations also demonstrated increased axonal and SCs regeneration within the reconstructed nerve gap in animals treated with the combined therapy. These results indicate that the functionalized SAP nanofiber scaffolds represented a promising biomaterial for application in peripheral nerve regeneration. And the combined therapy approach apparently promoted axonal regeneration and remyelination in the transected peripheral nerve.

## Data availability statement

The raw data supporting the conclusions of this article will be made available by the authors, without undue reservation.

## Ethics statement

The animal study was reviewed and approved by the Institutional Animal Investigation Committee of Beijing Luhe Hospital, Capital Medical University.

## Author contributions

LL and XSu: full access to all of the data in the study, take responsibility for the integrity of the data, and the accuracy of the data analysis. XSh, FQ, LL, and XSu: study concept and design. XSh, YP, SL, and SX: acquisition, analysis, or interpretation of data. XSh, FQ, and XSu: drafting of the manuscript. XSh, JL, and SL: statistical analysis. FQ and LL: study supervision. All authors: critical revision of the manuscript for important intellectual content.

## Funding

National Natural Science Foundation of China (31900968).

## Conflict of interest

The authors declare that the research was conducted in the absence of any commercial or financial relationships that could be construed as a potential conflict of interest.

## Publisher's note

All claims expressed in this article are solely those of the authors and do not necessarily represent those of their affiliated organizations, or those of the publisher, the editors and the reviewers. Any product that may be evaluated in this article, or claim that may be made by its manufacturer, is not guaranteed or endorsed by the publisher.
